# Robotik in der plastischen Chirurgie

**DOI:** 10.1007/s00104-022-01790-w

**Published:** 2023-01-10

**Authors:** Lisanne Grünherz, Epameinondas Gousopoulos, Carlotta Barbon, Semra Uyulmaz, Pietro Giovanoli, Nicole Lindenblatt

**Affiliations:** Klinik für Plastische Chirurgie und Handchirurgie, Universitätsspital Zürich, Rämistr. 100, 8091 Zürich, Schweiz

**Keywords:** Roboter, Robotisch assistierte Mikrochirurgie, Chirurgische Präzision, Rekonstruktive Lymphchirurgie, Zentrale Lymphchirurgie, Robotics, Robotic-assisted microsurgery, Reconstructive lymphatic surgery, Central lymphatic surgery, Surgical precision

## Abstract

In den letzten Jahren haben speziell für die Mikrochirurgie entwickelte Roboter das Anwendungsgebiet robotisch assistierter Operationen auch in der plastisch-rekonstruktiven Chirurgie erweitert. Derzeit gibt es zwei Mikrochirurgierobotersysteme, welche in der plastischen Chirurgie eingesetzt werden. Beide Systeme verfügen über Technologien zur Tremorreduzierung und Bewegungsskalierung, wodurch die Präzision und Geschicklichkeit des Chirurgen optimiert werden sollen. In der Klinik für Plastische Chirurgie und Handchirurgie des Universitätsspitals Zürich wird das Symani Surgical System® bereits bei vielen mikro- und supermikrochirurgischen Operationen, unter anderem bei autologen Brustrekonstruktionen, Nerventransfers und im Besonderen in der rekonstruktiven Lymphchirurgie, eingesetzt. Trotz spezieller technischer Herausforderungen, beispielsweise einem fehlenden haptischen Feedback, überwiegen nach entsprechendem Training des Mikrochirurgen die Vorteile, dazu zählen insbesondere kleinere Operationszugänge für anatomisch tiefe Strukturen und eine Verbesserung der chirurgischen Präzision.

**Video online:**

Die Onlineversion dieses Beitrags (10.1007/s00104-022-01790-w) enthält zwei ergänzende Videos.

## Abstract (en)

In recent years surgical robotic systems which were specifically developed for microsurgery have expanded the application of robotic-assisted surgery to plastic reconstructive surgery. Currently, there are two microsurgical robotic systems available for reconstructive plastic surgery. Both systems feature tremor reduction and motion scaling technologies, which are intended to optimize the surgeon’s precision and dexterity. In the Department of Plastic Surgery and Hand Surgery at the University Hospital Zurich, the Symani Surgical System® has already been used for many microsurgical and supermicrosurgical operations, including autologous breast reconstruction, nerve transfer and, in particular, reconstructive lymphatic surgery. Despite special technical challenges, such as a lack of haptic feedback, the advantages outweigh the disadvantages for an appropriately trained and skilled microsurgeon, including smaller surgical access incisions for anatomically deep structures and an improvement in surgical precision.

## Robotersysteme in der Mikrochirurgie

Robotisch assistierte Operationen wurden in den vergangenen Jahren als Routineverfahren in vielen chirurgischen Disziplinen erfolgreich implementiert. In der plastischen und rekonstruktiven Chirurgie erfolgte die erste roboterunterstützte mikrochirurgische Anastomose 2007 mit dem Da-Vinci-System – der Eingriff nahm noch über eine Stunde in Anspruch [16]. Seither steigen die Anwendungen von Robotersystemen auch in der plastischen Chirurgie stetig und reichen von mikrochirurgischen Eingriffen für autologe Brustrekonstruktionen bis hin zu supermikrochirurgischen Operationen im Rahmen der rekonstruktiven Lymphchirurgie [1, 6, 9, 13].

Dazu wurden Robotersysteme mit speziellem Fokus auf die Mikro- und Supermikrochirurgie entwickelt, wobei derzeit zwei Robotersysteme CE-zertifiziert sind und am Patienten Anwendung finden. Das Robotersystem MUSA (MicroSure, Eindhoven, Niederlande) wurde 2014 entwickelt und ist das erste verfügbare System seiner Art, welches bereits erfolgreich in präklinischen als auch in klinischen Studien eingesetzt wurde [17–19]. Das Robotersystem wird hierbei, inklusive seiner sog. Joysticks, am Operationstisch fixiert. Für die Operation wird der Roboter mit konventionellen Mikro- oder Supermikrochirurgieinstrumenten ausgestattet, was den Vorteil hat, dass das eigene Instrumentarium integriert werden kann und keine zusätzlichen Kosten anfallen.

Das zweite derzeit verfügbare System ist das Symani Surgical System® (Medical Microinstruments, Inc., Wilmington, DE, USA), welches in der Klinik für Plastische Chirurgie und Handchirurgie des Universitätsspitals Zürich weltweit erstmalig 2021 für die rekonstruktive Lymphchirurgie am Patienten eingesetzt wurde. Seitdem wurde das Symani Surgical System® bei vielen mikro- und supermikrochirurgischen Operationen erfolgreich verwendet [9].

Tremorreduzierung und Bewegungsskalierung optimieren Präzision und Geschicklichkeit

**Figure 1:**
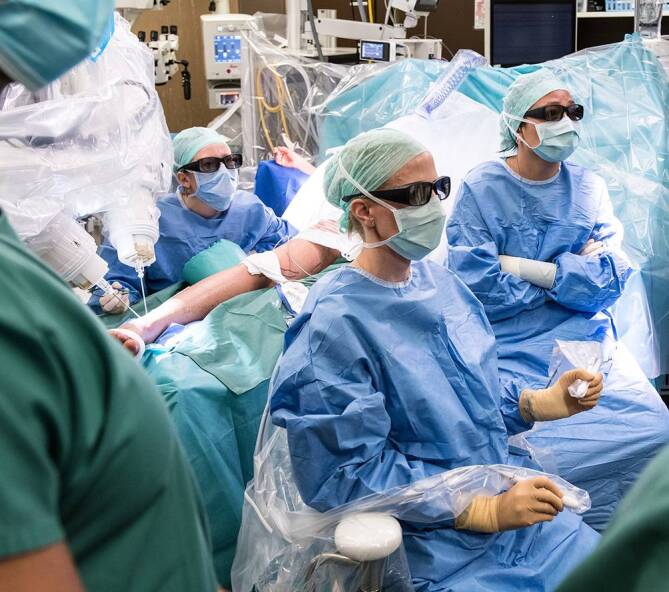

Das Symani Surgical System® verfügt über flexible Roboterarme, welche mit speziellen mikrochirurgischen Einweginstrumenten ausgestattet werden. Diese werden durch den Operateur mittels frei beweglicher Joysticks gesteuert, welche in der Handhabung den üblichen Mikroinstrumenten ähnlich sind (Pinzettengriff). Im Unterschied zu MUSA von MicroSure ermöglicht das Symani Surgical System® somit auch Teleoperationen, was insbesondere von Vorteil ist, wenn ein zweites Operationsteam parallel an einer anderen anatomischen Lokalisation operiert. Die Visualisierung des Operationssitus erfolgt entweder mit einem dreidimensionalen Exoskop bzw. Bildvisualisierungssystem oder mit einem Mikroskop (Abb. 1). Letzteres hat den Vorteil, dass ein zweiter Chirurg mit Mikroinstrumenten assistieren kann und erlaubt zudem einen schnellen Wechsel auf ein konventionelles manuelles Vorgehen. Sowohl das Symani Surgical System® als auch der MUSA verfügen über Technologien zur Tremorreduzierung und Bewegungsskalierung, wodurch die Präzision und Geschicklichkeit des Chirurgen optimiert werden sollen.

## Anwendungsgebiete in der Lappenchirurgie

Robotisch assistierte Operationen werden bereits in einigen Bereichen der plastisch-rekonstruktiven Chirurgie mit dem Ziel kleinerer Narben und einer möglichst atraumatischen Dissektion durchgeführt.

Das Da-Vinci-System wurde hierbei insbesondere im Rahmen der autologen Brustrekonstruktion mittels „Deep-inferior-epigastric-perforator“(DIEP)-Lappen eingesetzt [4]. In diesem Kontext konnte gezeigt werden, dass der Einsatz eines Roboters eine minimal-invasive intraabdominale Dissektion des Gefäßpedikels ermöglicht, wodurch der Faszienschnitt auf 1,5–3 cm reduziert und eine Pedikellänge von 10–15 cm erreicht werden konnte [13]. Zudem ermöglicht die Verwendung eines Robotersystems einen vollständig extraperitonealen Zugang [5]. Angesichts der bekannten Morbidität der abdominalen Spenderstelle sind dies sehr attraktive Ansätze zur Verbesserung der Ergebnisse nach der DIEP-Lappenentnahme [8].

Robotersysteme eignen sich für freie Lappenplastiken und epineurale Koaptationen

Des Weiteren hat sich das Da-Vinci-System für transorale Zugänge im Rahmen von Tumorresektionen und Rekonstruktionen im Oropharynx, beispielsweise mittels Radialislappen oder „anterolateral thigh flap“, bewiesen. Durch den Einsatz eines Robotersystems kann hierbei eine Mandibulotomie umgangen und die Morbidität des Eingriffes somit signifikant gesenkt werden [14].

**Figure 2:**
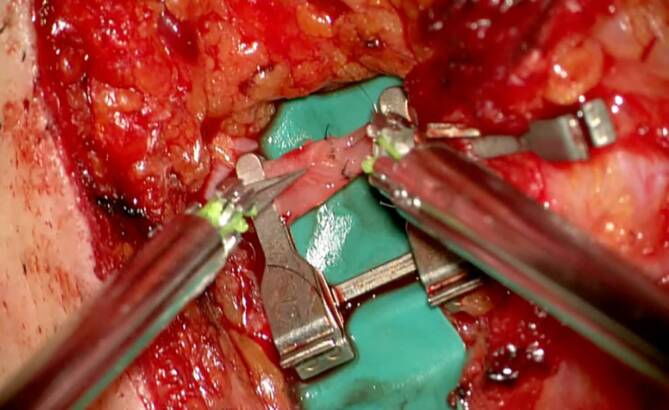

In unserer Klinik haben wir das Symani Surgical System® bereits erfolgreich für mikrochirurgische Anastomosen bei verschiedenen freien Lappen, unter anderem „profunda artery perforator (PAP) flap“ und „superficial circumflex iliac artery perforator (SCIP) flap“ sowie neurovaskulärer Grazilislappen zur Gesichtsreanimation, eingesetzt (Abb. 2). Zudem konnten wir zeigen, dass das Robotersystem auch für die epineurale Koaptation im Rahmen von Nervenrekonstruktionen eingesetzt werden kann [9].

## Robotisch assistierte rekonstruktive Lymphchirurgie

Die rekonstruktive Lymphchirurgie, welche höchste Präzision und Geschicklichkeit voraussetzt, stellt derzeit eines der Hauptanwendungsgebiete für Roboter in der plastischen Chirurgie dar. Van Mulken und sein Team haben 2020 die ersten robotisch assistierten lymphovenösen Anastomosen (LVAs) mit Micro Sure’s MUSA bei Patienten mit Armlymphödem durchgeführt [19]. Das Symani Surgical System® hingegen wurde erstmals durch uns für die rekonstruktive Lymphchirurgie eingesetzt [9]. Neben der Durchführung von LVAs bei Patienten mit Lymphödem sowie lympholymphatischen Anastomosen zur Behandlung von Lymphfisteln hat sich das Symani Surgical System® auch für den mikrochirurgischen Anschluss des Lymphknotenlappens bewehrt (Video 1 s. Zusatzmaterial online). Hierbei ermöglicht das Symani Surgical System® anatomisch tief gelegene Strukturen, trotz kleiner Inzisionen, gut zu erreichen. Im Rahmen einer Studie haben wir zudem bei insgesamt 31 Anastomosen den Trainingseffekt in Bezug auf den Zeitunterschied zwischen manuellen und robotisch assistierten Anastomosen analysiert. Dabei konnten wir eine steile Lernkurve beobachten, wobei sich nach entsprechendem Training des Mikrochirurgen eine signifikante Reduktion in der Operationszeit für robotisch assistierte LVAs zeigte und die zeitliche Differenz zwischen manuellen und robotisch assistierten Anastomosen nur noch geringfügig war ([3]; Video 2 s. Zusatzmaterial online).

Ein weiteres Anwendungsgebiet in der Lymphchirurgie stellt die robotisch assistierte Entnahme des Omentumlappens dar, welcher als Lymphgewebslappen an die vom Lymphödem betroffene Extremität transplantiert wird. Die robotergestützte Entnahme bietet eine unvergleichbare Visualisierung des Gewebes und ermöglicht so eine sehr präzise Gewebedissektion und Pedikelpräparation. Darüber hinaus wird das Risiko, benachbarte anatomische Strukturen zu verletzen, aufgrund der Tremorreduzierung und der größeren Bewegungsfreiheit minimiert. Die Einbeziehung zusätzlicher bildgebender Verfahren, wie z. B. einer fluoreszierenden Optik zur Visualisierung der Blut- und Lymphgefäßmuster, ermöglicht zudem eine Verbesserung des Lappendesigns und der Entnahme. Trotz der Verlängerung der Operationszeit im Vergleich zur laparoskopisch assistierten Chirurgie stellt die robotergestützte Entnahme einen vielversprechenden Ansatz für die Lymphknotenentnahme dar [7, 12].

## Zentrale Lymphchirurgie

Auch im Rahmen der zentralen Lymphchirurgie wird der Einsatz von Robotern zukünftig Operationen an anatomisch tief gelegenen Strukturen vereinfachen. Dies ist z. B. bei seltenen zentralen lymphatischen Anomalien (CCLA) oder iatrogenen Läsionen des Ductus thoracicus der Fall. Bei diesen sehr seltenen Erkrankungen kommt es durch den ständigen Austritt von Chylus zu einem permanenten Protein- und Flüssigkeitsverlust, welcher wiederum Infektionen und weitere Komplikationen begünstigt und zu einer Mortalität von bis zu 50 % führt [20].

Robotersysteme sind insbesondere in tiefer gelegenen Regionen des Körpers von Vorteil

**Figure 3:**
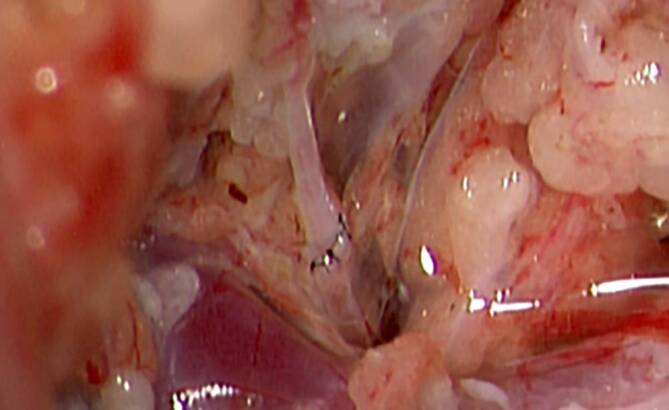

Bei erfolgloser konservativer Therapie besteht die Möglichkeit, eine Ductus-thoracicus-Venen-Anastomose durchzuführen, welche wir in der Plastischen Chirurgie und Handchirurgie des Universitätsspitals Zürich bereits bei einigen Erwachsenen sowie auch Kindern (Abb. 3) durchgeführt haben [10, 11]. Im Gegensatz zu einer interventionell-radiologischen Embolisation ermöglicht dies eine Rekonstruktion des physiologischen Lymphabflusses [6–8]. Aufgrund der Lage des Ductus thoracicus im Retroperitoneum respektive im hinteren Mediastinum werden ein verhältnismäßig großer Zugangsweg sowie spezielle Mikrochirurgieinstrumente benötigt. In diesem Kontext würde der Einsatz eines Roboters eine kleinere Inzision ermöglichen und den Zugangswegs bedeutend vereinfachen.

## Lymphchirurgie bei Kindern

**Figure 4:**
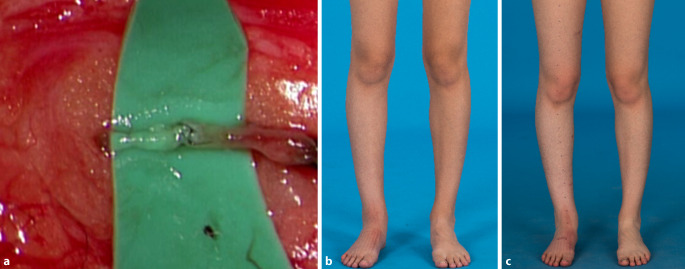

In den vergangenen Jahren findet die rekonstruktive Lymphchirurgie zunehmend auch bei Kindern mit Lymphödem oder zentralen lymphatischen Anomalien Anwendung [11, 15, 20]. So haben wir kürzlich unter anderem bei einem 14-jährigen Jungen mit primärem Lymphödem der proximalen Oberschenkel sowie des Penis einen Lymphgewebetransfer vom Omentum majus zu beiden Leisten sowie die Anlage von LVAs auf dem Dorsum penis durchgeführt. Ebenso konnte bei einer 17-jährigen Patientin mit sekundärem Lymphödem des Beines nach Lymphadenektomie der Leiste durch multiple lymphovenöse Anastomosen eine signifikante Reduktion des Lymphödems erreicht werden (Abb. 4). Dadurch konnte eine Rückbildung des Ödems sowie eine Schmerzfreiheit bei körperlicher Betätigung erreicht werden. Bedenkt man, dass kindliche Narben einem ständigen Wachstum ausgesetzt sind und dadurch tendieren, breit zu werden, sollte bei Kindern ganz besonders auf kleine Inzisionen geachtet werden. Solche Eingriffe stellen somit ein weiteres vielversprechendes Einsatzgebiet für robotisch assistierte Operationen dar.

## Aktuelle Herausforderungen

Zu den aktuellen Herausforderungen bei der Integration der Robotertechnologie zählt im Besonderen die längere Operationszeit. Obwohl in mehreren Studien gezeigt werden konnte, dass die Lernkurve steil verläuft und die Häufigkeit des Übens sowie das Niveau der mikrochirurgischen Erfahrung einen weiteren positiven Einfluss haben, benötigt die robotisch assistierte Anastomose bis dato noch mehr Zeit [2, 16, 18]. Im Hinblick auf die Zeiteffizienz sollte das gesamte Operationsteam entsprechend geschult werden, um einem zusätzlichen Zeitverlust entgegenzuwirken.

Eines der Hauptnachteile der Robotertechnologie stellt aktuell das Fehlen eines haptischen Feedbacks und die Notwendigkeit für den ausführenden Chirurgen, während der Durchführung der Anastomose ein „See-feel“-Konzept zu entwickeln, dar. Durch den Einsatz geeigneter bildgebender Verfahren lässt sich dies zwar zum Teil verbessern, zukünftig könnte jedoch die Integration zusätzlicher Biosensoren das haptische Feedback verbessern und somit die chirurgische Präzision und die atraumatische Handhabung nochmals deutlich optimieren [9, 18].

## Schlussfolgerung

Aktuell findet die Robotertechnologie in der plastischen Chirurgie vor allem in der rekonstruktiven Lymphchirurgie Anwendung. Aufgrund der Zeiteffizienz der Operation und des Fehlens eines haptischen Feedbacks sollten robotisch assistierte Operationen allerdings erfahrenen sowie entsprechend geschulten Mikrochirurgen vorbehalten bleiben. Mit der stetigen Verbesserung der Technologie und dem Vorteil kleinerer Operationszugänge, insbesondere für anatomisch tiefer gelegene Strukturen, werden Roboter in der plastisch-rekonstruktiven Chirurgie zukünftig sehr wahrscheinlich vermehrt Anwendung finden.

## Fazit für die Praxis

- Derzeit finden zwei speziell entwickelte Robotersysteme, Microsure’s MUSA® und das Symani Surgical System®, in der mikrochirurgischen Anwendung.
- Robotersysteme werden insbesondere im Rahmen der autologen Brustrekonstruktion und rekonstruktiven Lymphchirurgie eingesetzt.
- Zu den aktuellen Herausforderungen zählen eine längere Operationszeit und das Fehlen eines haptischen Feedbacks.
- Bei entsprechendem Training mit dem Roboter überwiegen die Vorteile, u. a. kleinere Operationszugänge für anatomisch tiefe Strukturen und eine Verbesserung der chirurgischen Präzision.

## Supplementary Information






## References

[1] Aitzetmuller MM, Klietz ML, Dermietzel AF, Hirsch T, Kuckelhaus M (2022). Robotic-assisted microsurgery and its future in plastic surgery. J Clin Med.

[2] Alrasheed T, Liu J, Hanasono MM, Butler CE, Selber JC (2014). Robotic microsurgery: validating an assessment tool and plotting the learning curve. Plast Reconstr Surg.

[3] Barbon C, Grünherz L, Uyulmaz S, Giovanoli P, Lindenblatt N (2022). Exploring the learning curve of a new robotic microsurgical system for microsurgery. JPRAS Open.

[4] Bishop SN, Asaad M, Liu J, Chu CK, Clemens MW, Kapur SS (2022). Robotic harvest of the deep inferior epigastric perforator flap for breast reconstruction: a case series. Plast Reconstr Surg.

[5] Choi JH, Song SY, Park HS, Kim CH, Kim JY, Lew DH (2021). Robotic DIEP flap harvest through a totally extraperitoneal approach using a single-port surgical robotic system. Plast Reconstr Surg.

[6] Dobbs TD, Cundy O, Samarendra H, Khan K, Whitaker IS (2017). A systematic review of the role of robotics in plastic and reconstructive surgery-from inception to the future. Front Surg.

[7] Frey JD, Yu JW, Cohen SM, Zhao LC, Choi M, Levine JP (2020). Robotically assisted omentum flap harvest: a novel, minimally invasive approach for Vascularized lymph node transfer. Plast Reconstr Surg Glob Open.

[8] Lindenblatt N, Gruenherz L, Farhadi J (2019). A systematic review of donor site aesthetic and complications after deep inferior epigastric perforator flap breast reconstruction. Gland Surg.

[9] Lindenblatt N, Grunherz L, Wang A, Gousopoulos E, Barbon C, Uyulmaz S (2022). Early experience using a new robotic microsurgical system for lymphatic surgery. Plast Reconstr Surg Glob Open.

[10] Lindenblatt N, Gutschow CA, Vetter D, Puippe G, Broglie Däppen M, Schneiter D (2021). Lympho-venous anastomosis for the treatment of congenital and acquired lesions of the central lymphatic system: a multidisciplinary treatment approach. Eur J Plast Surg.

[11] Lindenblatt N, Puippe G, Broglie MA, Giovanoli P, Grunherz L (2020). Lymphovenous anastomosis for the treatment of thoracic duct lesion: a case report and systematic review of literature. Ann Plast Surg.

[12] Ozkan O, Ozkan O, Cinpolat A, Arici C, Bektas G, Can Ubur M (2019). Robotic harvesting of the omental flap: a case report and mini-review of the use of robots in reconstructive surgery. J Robot Surg.

[13] Selber JC (2020). The robotic DIEP flap. Plast Reconstr Surg.

[14] Selber JC (2010). Transoral robotic reconstruction of oropharyngeal defects: a case series. Plast Reconstr Surg.

[15] Taghinia AH, Upton J, Trenor CC, Alomari AI, Lillis AP, Shaikh R (2019). Lymphaticovenous bypass of the thoracic duct for the treatment of chylous leak in central conducting lymphatic anomalies. J Pediatr Surg.

[16] van der Hulst R, Sawor J, Bouvy N (2007). Microvascular anastomosis: is there a role for robotic surgery?. J Plast Reconstr Aesthet Surg.

[17] van Mulken TJM, Boymans C, Schols RM, Cau R, Schoenmakers FBF, Hoekstra LT (2018). Preclinical experience using a new robotic system created for microsurgery. Plast Reconstr Surg.

[18] van Mulken TJM, Schols RM, Qiu SS, Brouwers K, Hoekstra LT, Booi DI (2018). Robotic (super) microsurgery: Feasibility of a new master-slave platform in an in vivo animal model and future directions. J Surg Oncol.

[19] van Mulken TJM, Schols RM, Scharmga AMJ, Winkens B, Cau R, Schoenmakers FBF (2020). First-in-human robotic supermicrosurgery using a dedicated microsurgical robot for treating breast cancer-related lymphedema: a randomized pilot trial. Nat Commun.

[20] Weissler JM, Cho EH, Koltz PF, Carney MJ, Itkin M, Laje P (2018). Lymphovenous anastomosis for the treatment of chylothorax in infants: a novel microsurgical approach to a devastating problem. Plast Reconstr Surg.

